# Improving Cavalieri volume estimation based on non‐equidistant planar sections: The trapezoidal estimator

**DOI:** 10.1111/jmi.13141

**Published:** 2022-09-28

**Authors:** Mads Stehr, Markus Kiderlen, Karl‐Anton Dorph‐Petersen

**Affiliations:** ^1^ Department of Finance Copenhagen Business School Frederiksberg Denmark; ^2^ Department of Mathematics Aarhus University Aarhus Denmark; ^3^ Translational Neuropsychiatry Unit, Department of Clinical Medicine Aarhus University Aarhus Denmark; ^4^ Translational Neuroscience Program, Department of Psychiatry University of Pittsburgh Pittsburgh Pennsylvania USA

**Keywords:** asymptotic variance, Cavalieri estimator, dropouts, Newton–Cotes estimation, perturbed systematic sampling, stereology, trapezoidal estimator

## Abstract

The Cavalieri estimator allows one to infer the volume of an object from area measurements in equidistant planar sections. It is known that applying this estimator in the non‐equidistant case may inflate the coefficient of error considerably. We therefore consider a newly introduced variant, the trapezoidal estimator, and make it available to practitioners. Its typical variance behaviour for natural objects is comparable to the equidistant case. We state this unbiased estimator, describe variance estimates and explain how the latter can be simplified under rather general but realistic models for the gaps between sections. Simulations and an application to a synthetic area function based on parietal lobes of 18 monkeys illustrate the new methods.

## 1 INTRODUCTION

The purpose of this paper is to summarize state‐of‐the‐art results concerning volume estimation of spatial objects using Cavalieri‐type estimates and make them available to applications in microscopy. In contrast to the classical setting, slices of exactly the same thickness are not always realistic in applications – especially when estimating from thick tissue slabs, and we explain why the new, unbiased *trapezoidal estimator* has better variance behaviour in this setting than the existing alternatives. We also describe how the variance of the trapezoidal estimator can be estimated in different realistic scenarios and that it even can be used if dropouts occur. Part of this paper serves as a more practitioner‐oriented description of the mathematically rigorous results of papers 1, 2, 3, whereas in particular the formulas for variance estimation are novel.

The quantity of interest is the volume *Q* of some non‐random and bounded object $Y\subseteq R^{3}$. Traditionally, this quantity is estimated from parallel planar sections placed systematically with a fixed distance *T* apart: With ${\left\{S_{k}\right\}}_{k=0}^{N}$ denoting the cross sections of *Y* and ${\left\{\mathrm{Area}\left(S_{k}\right)\right\}}_{k=0}^{N}$ their associated known areas, the Cavalieri volume estimator of *Q* is

(1) $$\hat{Q}=T\sum_{k=0}^{N}\mathrm{Area}\left(S_{k}\right).$$

The (starting) position of the stack of parallel planar sections is randomly chosen along some convenient sampling axis, turning $\hat{Q}$ into a random variable. If the starting position is uniform in an interval of length *T*, the estimator $\hat{Q}$ is in fact unbiased, that is $E\hat{Q}=Q$, and its variance, given as a function of the slice thickness *T*, is described in great detail in, for instance, Ref. 4. As the terminology is not standardized across different research communities, we explain the following conventions that will be followed throughout the paper: A mathematical plane of thickness zero will be referred to as *section plane*, or simply a *section*. A *slice* between two consecutive sections can refer to either a *slab* (with a typical thickness in mm) or a *histological section* (typical thickness in μm).

The formulation of the estimator in (1) relies on a number of idealizing and simplifying assumptions. One of them is that the profile areas are known exactly, although they are often determined by systematic subsampling in the section. This additional source of errors contributes to the total variance of (1), as was outlined and quantified in [Ref. 5, Section 6] and Refs. 6, 7; see also the references given therein. Following the lines of arguments described in these papers, one could – based on the law of total variance – easily extend the methods to area measurements with errors. This is, however, not the purpose of the present work. Another underlying assumption is that the sections are equidistant, or, in other words, that the slices all have exactly the same thickness. In particular in the case of physical sections, deviations from exact equidistant section positions might occur. Traditionally, this imprecision has been considered as inconsequential and thus been ignored, and the estimator (1) was applied with *T* now being the *average* distance between two consecutive section planes. Such an estimator, usually called *generalized Cavalieri estimator*, is again unbiased [Ref. 8, Theorem 1], however, as suggested by Baddeley et al.^8^ and quantified by Ziegel et al.,^9^, ^10^ the variance of the estimator may explode compared to the equidistant case; the exact behaviour is explained in the following section.

**FIGURE 1:**
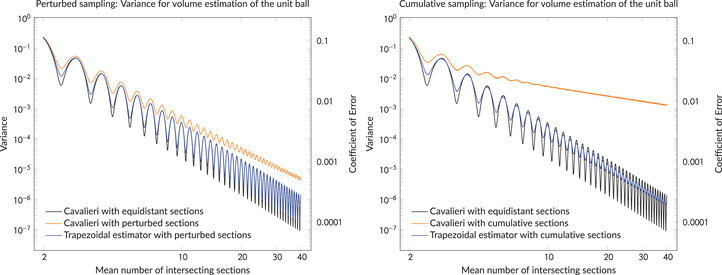
Variances for volume estimation of the three‐dimensional ball with radius 1. Both figures show the variance of (1) based on equidistant sections, the variance of (1) based on non‐equidistant sections and the variance of the trapezoidal estimator based on non‐equidistant sections. In the figure on the left, the positions of the non‐equidistant section planes are generated by independent perturbations of equidistant section positions, with the magnitude of the perturbations being such that the average relative deviation of the distance from *T* is 5%. In the figure on the right, the positions of the non‐equidistant section planes are generated by accumulating independent increments, with the magnitude of the increments chosen such that the average relative deviation is 5%

Figure 1 illustrates this problem in the case of volume estimation for the three‐dimensional ball of radius 1. On a log –log  scale the figures show, as a function of the mean number of hitting section planes (that is, 2/T), the variance of (1) under equidistant and two examples of non‐equidistant sampling. Not only is the variance from either type of non‐equidistant sampling always larger than in the classical case, the aggravation is also serious, and the variance in Figure 1 (left) is already doubled when a mean number of 2/T=5.3 sections are taken. In the cumulative model, to be defined later in this section and depicted in Figure 1 (right), the variance of the Cavalieri estimator behaves even worse, as it decreases at a much slower rate than the other variances. Figure 1 also indicates that the variance still decreases to zero as the mean number of non‐equidistant section planes grows, but at a rate that is clearly slower than in the equidistant case.

To ameliorate this situation, we suggest to use a new estimator, the so‐called *trapezoidal estimator*, first defined in Ref. 1. This estimator requires slightly more input data than the classical Cavalieri estimator, but it is again unbiased and its variance, also depicted in Figure 1, behaves essentially like the variance of the Cavalieri estimator with equidistant sections and thus eliminates the problem of unequal spacing.

**FIGURE 2:**
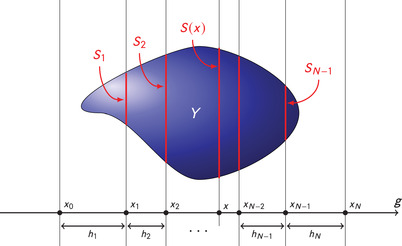
Sampling of a three‐dimensional object *Y* with parallel section planes (appearing as lines) that all are orthogonal to the sampling axis *g*. The plane at position *x* has section profile S(x), and $S_{k}=S\left(x_{k}\right)$

Besides the section areas $\left\{\text{Area}\left(S_{k}\right)\right\}$ the new estimator also requires the distances of all (randomly located) section planes from their neighbours. More precisely, for k=1,…,N let $h_{k}$ denote the thickness of the *k*th slice, that is, the distance of the section planes containing the profiles $S_{k-1}$ and $S_{k}$, respectively (see Figure 2). The trapezoidal estimator takes the form

(2) $${\hat{Q}}_{1}=\sum_{k=1}^{N}\frac{h_{k}+h_{k+1}}{2}\mathrm{Area}\left(S_{k}\right).$$

The results in the present paper merely require that the random thicknesses $h_{k}$ arise as increments from a translation‐invariant random set of sampling points, meaning intuitively that the locations of the sections $S_{0},\cdots ,S_{N}$ do not oversample or undersample any position. This is in particular satisfied, if the sections are equidistant with a uniform starting position as described after (1), but it also allows for correlated gap lengths. As indicated in Figure 2, this estimator (and in fact also the Cavalieri estimator (1)) uses the fact that the object *Y* is strictly contained between the two section planes *S*_0_ and $S_{N}$, respectively, and consequently $\mathrm{Area}\left(S_{0}\right)=\mathrm{Area}\left(S_{N}\right)=0$. It also requires that all individual thicknesses of slices between these two planes are known. This is certainly true in the equidistant case, where we have $h_{k}=T$ for all *k*. In this case, the trapezoidal estimator coincides with the Cavalieri estimator.

**FIGURE 3:**
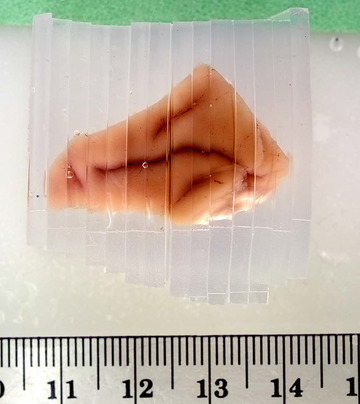
Agar embedded monkey parietal lobe cut into 2.5‐mm slabs perpendicular to the intra‐parietal sulcus. The position of the individual cuts are easily visible in the image. Ruler included for reference. The original image was approximately 2000 pixels wide. The total thickness of the stack of slabs was measured precisely using a digital caliper. Notice the variation in slab thickness. The small gap between slabs 7 and 8 in the picture was taken into account when assessing the cut positions. Photograph by Glenn Konopaske and Ruth Henteleff, preparation as described in Ref. 11

Non‐equidistant sections can occur due to a number of reasons depending upon how the cuts are made. Traditionally, stereological studies were based upon exhaustive sectioning of the tissue into histological sections, a systematic subsample of which was used for the volume estimation using the Cavalieri estimator. The standard devices for generating μm‐thin histological serial sections (microtome, cryostat or vibratome) typically cut sections with very small errors. The exact errors of these individual sections and their exact positions within the tissue are not obtainable in any practical way in a standard lab setting. These errors are expected to fit the perturbed model (see later), and are likely small and insignificant. However, many newer stereological designs use a two‐step sampling procedure where the tissue is cut into mm to cm thick slabs, from the faces of which, a few μm‐thick histological sections may subsequently be cut. This saves sectioning time and allows for improved storage of tissue as slabs for future use (see, e.g. Refs. 12, 13). Also, some designs use the tissue between the sampled sections for further subsampling (see, e.g. Refs. 14, 15). The initial slabs are typically cut with visible errors in position. This may be due to unavoidable human error when cutting by hand using a guide, due to compression/elasticity of the sectioned object (organ/lump of tissue) or due to drift of the thin blade(s) while sectioning in various cutting devices. The positions (and errors in these) of the individual cuts are easily observable in high‐quality photos of the edge of the stack of the cut slabs (see Figure 3). This is simple to implement in a standard laboratory setting. Including a ruler in the picture as in Figure 3 allows for measurements in the picture. The precision can be improved by measuring the total thickness of the stack of slabs using a digital caliper. This is typically done anyway to calculate the mean slab thickness.

Although no model assumptions are needed, estimating the variance of ${\hat{Q}}_{1}$ becomes more robust when one of the two common sampling models hold. We describe them informally and refer to Definitions 1 and 2 in Ref. 3 for a mathematically rigorous exposition. We emphasize that no model assumptions are needed to compute the trapezoidal estimator ${\hat{Q}}_{1}$, and similarly its variance can be estimated without such assumptions (see Definition 1). If the correct model is known, this can be exploited using a more specific variance estimator; cf.  Definitions 2 and 3.

### Perturbed model

We sample from the perturbed model if the positions of the section planes are found by independent perturbations of equidistant section positions. This means that the actual location of each cut deviates from its intended position by a small random displacement. Examples of this are the use of various devises for cutting agar embedded tissue into uniform slabs, for example: (1) Macrotome [Ref. 16, fig. 24], where a knife mounded to a tread cuts the agar embedded tissue at systematic positions; (2) Array of razorblades [Ref. 17, fig. 7]; (3) ‘Shoebox cutter’, where a brick of agar with tissue and a mm paper is cut using a cutting guide, see Ref. 18 and [Ref. 15, fig. 3E–F]. These methods generate equidistant intended positions for cutting. However, the knife may drift for each cut, independent of the previous cut.

If the perturbations are degenerate and constant 0, the resulting positions are equidistant.

### Cumulative model

We sample from the cumulative model if the distances between consecutive section planes are independent and have the same distribution. An example of this is when the specimen is repeatedly pushed against a firm plate before cutting, where the position of the knife is fixed relative to the plate. This is the case in a standard kitchen bread or meat slicer. Also, this is the intuitive way a human cuts unguided when aiming by free hand at a certain slab thickness – for example, when cutting 1 cm bread slices in the kitchen.

The Cavalieri estimator (1) behaves much worse under cumulative sampling than under perturbed sampling.^9^ It turns out, however, that (1) is inferior to the trapezoidal estimator in both cases. In fact, as shown in the next section, the Cavalieri estimator cannot exploit the smoothness of the area function f(x)=Area(S(x)) (cf. Figure 2) under cumulative sampling, and only to some degree under perturbed sampling, whereas the trapezoidal estimator is designed to do so to a much larger degree.

Non‐equidistant sections cannot only occur due to an initial non‐equidistant sampling mechanism but also due to dropouts. These are sections that got lost in the preparation process or for which the profile area cannot be determined, for example, when the staining is failing or when a histological section is lost due to tears and folds during the processing. Typically, a new histological section will be generated. However, this is not always possible – especially in archival material.

Since the trapezoidal estimator takes the observed slice thicknesses into account, its order of variance is independent of whether or not dropouts have occurred. This property is in strong contrast to the Cavalieri estimator. If dropouts have happened and the initial section distances are known (that is, also for the section planes without measurable area), Ref. 10 suggest an alternative estimator to (1) in which missing area measurements are approximated by an average of known neighbouring section‐areas. The variance of this method is smaller than that of (1), but it is generally significantly higher than in the case of equidistant sections; see [Ref. 9, Propositions 3 & 4] and [Ref. 10, Propositions 3–5]. Moreover, this method is also inferior to the trapezoidal estimator, which can be applied without any additional approximation procedure. In this and the above‐mentioned papers, dropouts are modelled by independent thinning where each section is independently dropped with a given probability.

## 2 VOLUME ESTIMATORS AND THEIR VARIANCE BEHAVIOUR

In this section, we give a formal description of the variance behaviour of the trapezoidal estimator. Recall that the trapezoidal estimator actually coincides with the Cavalieri estimator when the sections are equidistant, and thus the results presented here also hold for the classical case of Cavalieri estimation based on equidistant sampling. To give a proper variance representation, we need a slightly more mathematical approach.

Recall that $Y\subseteq R^{3}$ denotes the object of interest, and that we observe a stack of parallel cross sections $S_{0},S_{1},\text{…}$, $S_{N}$ of *Y* with associated known areas $\mathrm{Area}\left(S_{0}\right),\mathrm{Area}\left(S_{1}\right),\text{…},\mathrm{Area}\left(S_{N}\right)$, where the first and last of them are zero. For the trapezoidal estimator, we need the locations of the sections relative to each other, that is, the slice thicknesses $h_{1},h_{2},\text{…},h_{N}$. However, for the descriptions in this and the following section, it will be convenient to also consider the actual locations $x_{0}<x_{1}<\cdots <x_{N}$ of the sections along the sampling axis. By this we mean that at a position $x_{k}\in R$ (k=0,⋯,N) the plane orthogonal to the sampling axis has intersection $S_{k}$ with *Y*; see Figure 2. As indicated in Figure 2, we assume that the object *Y* is strictly contained in the strip between the two planes at *x*_0_ and at $x_{N}$, so these two planes do not hit *Y*.

The area f(x)=Area(S(x)) of the intersection profile of *Y* with a plane at position *x*, as visualized in Figure 2, gives rise to the so‐called *area function* *f*. This function is zero to the left of *x*_0_ and to the right of $x_{N}$. By a Cavalieri‐type argument, the volume *Q* of *Y* is nothing else than $\int_{R}f\left(x\right)dx$. Cavalieri estimation is thus solving the problem of estimating the integral $Q=\int_{R}f\left(x\right)dx$ from finitely many values of *f* at random sampling points. This mathematical formulation of the problem is used in the papers 1, 2, 3, which we will repeatedly refer to.

**FIGURE 4:**
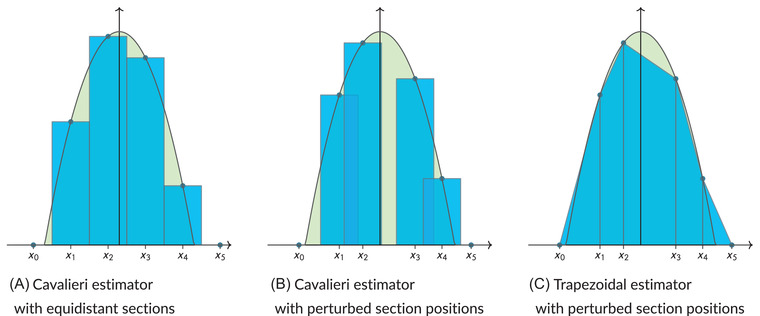
Illustration of the approximation schemes used to construct the Cavalieri estimators (under equidistant and perturbed sampling, respectively) and the trapezoidal estimator. The depicted area function relating to the ball of radius 1 is given by $f\left(x\right)=\pi \left(1-x^{2}\right)$ for x∈[−1,1]. The green area is *Q* and the combined areas of the blue polygons are the estimates of *Q*

Considering the estimation as a numerical integration problem, the fact that the trapezoidal estimator (2) outperforms the Cavalieri estimator (1) under non‐equidistant sampling is not that surprising. The Cavalieri estimator simply approximates the integral of *f* by a naive weighted sum, which coincides with a Riemann sum in the equidistant case. This approximation is too crude when points are not equidistant. In contrast, the trapezoidal estimator utilizes the actual sampling locations to construct a better approximation of the function *f*: On the interval $\left[x_{k},x_{k+1}\right]$ between two consecutive sampling points, the trapezoidal rule approximates the area function *f* by a linear function, which leads to an approximation of the integral $\int_{x_{k}}^{x_{k+1}}f\left(x\right)dx$ by

(3) $$\begin{array}{ccc} \int_{x_{k}}^{x_{k+1}}f\left(x\right)dx & \approx & \frac{f\left(x_{k+1}\right)+f\left(x_{k}\right)}{2}\left(x_{k+1}-x_{k}\right)\\ & & =\frac{\mathrm{Area}\left(S_{k+1}\right)+\mathrm{Area}\left(S_{k}\right)}{2}h_{k+1}, \end{array}$$

where it has been used that $x_{k+1}-x_{k}=h_{k+1}$ is the (random) distance between the sections at positions $x_{k}$ and $x_{k+1}$. Summing over all these integral approximations yields the trapezoidal estimator (2). Figure 4 gives a complete illustration of the estimators in the case of volume estimation for the three‐dimensional ball of radius 1. In this case, the area function is $f\left(x\right)=\pi \left(1-x^{2}\right)$ for x∈[−1,1].

The precision of all the above‐mentioned estimators is determined by the smoothness of the area function *f* associated to *Y*: For an integer *m*, we say that *f* is *weakly* (*m*, 1)*‐piecewise smooth* if it has continuous derivatives up to order m−1, if the derivative of order *m* has a finite number of finite jumps, and if the derivative of order m+1 has a finite number of possibly infinite jumps. This assumption is slightly less restrictive than the traditional smoothness concept considered in, for example, Refs. 4, 9, 10 and Ref. 2 of (*m*, 1)‐piecewise smoothness, which additionally requires that the derivative of order m+1 must in fact have finite jumps. However, in Ref. 3 it is argued that the results in all of the above‐mentioned literature also holds true for *weakly* (*m*, 1)‐piecewise smooth functions.

**FIGURE 5:**
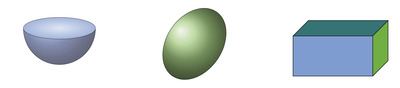
The smoothness of the area function depends on the orientation of the sampling axis with respect to the object. The hemisphere on the left is 1‐oriented if the sampling axis is orthogononal to the *z*‐axis, but it is 0‐oriented if the sampling axis is parallel to the *z*‐axis. The ellipsoid in the middle is 1‐oriented for all sampling axes. The box to the right is 0‐oriented if the sampling axis is parallel to one of the coordinate axes, it is 1‐oriented if the sampling axis is orthogonal to an edge (excluding the cases where it is parallel to a coordinate axis), and in all other cases it is 2‐oriented

The order of smoothness *m* determines the decrease rate of the variance of the estimators, and for this reason it is desirable to maximize *m*. For instance, if a convex three‐dimensional object has a smooth boundary, the values m=0 and m=1 are very common, and we will restrict considerations to these smoothness cases in the present paper, see also the discussion in the paragraph directly after Theorem 1. As the smoothness properties of the object *Y* cannot be changed, the smoothness *m* of the area function can only be influenced by a careful choice of the sampling axis. To simplify notation, we say that *Y* is *m* *‐oriented (with respect to the sampling axis)* if the area function *f*, based on this axis, is weakly (*m*, 1)‐piecewise smooth. In Ref. 6, such a set *Y* is called ‘object‐*m*’ (though requiring the slightly stronger (*m*, 1)‐piecewise smoothness), but our diction emphasizes that this property depends on both, *Y* and the sampling axis. We give some examples to illustrate this in practical terms. Assume that *Y* contains a ‘flat’ part in its boundary, for instance if *Y* is a cylinder or a hemisphere. If the sampling planes are parallel to this flat part of *Y*, then *Y* is always 0‐oriented (see Figure 5). Although atypical in biological applications, consider for illustration the particular case where *Y* is a *polytope*, meaning that all its sides are ‘flat’ like for a cube, a pyramid or an ideal crystal. If the sampling axis is orthogonal to one side of *Y* – and thus the sampling planes are all parallel to this side – the object *Y* is 0‐oriented with respect to the sampling axis. If the sampling axis is orthogonal to one of the edges of *Y* (i.e. sampling planes are parallel to this edge) then *Y* is 1‐oriented. All other axes turn the polytope into a 2‐oriented object.

On the other hand, a convex object *Y* that has a smooth boundary (with positive curvature everywhere) is typically 1‐oriented. To consider an even more specialized case: an ellipsoid *Y* is a 1‐oriented object with respect to any axis.

Summarizing, when choosing the sampling axis, orientations that lead to section planes parallel to a flat side of the boundary of *Y* must be avoided in order to get the best variance behaviour. If this is not possible, one has to apply the theory below with m=0. Otherwise, m=1 is often an appropriate choice.

The following result provides a basic characterization of the variance of the trapezoidal estimator based on slices with an average thickness *T*; see [Ref. 2, Proposition 6.1] for details. In principle, the statement about the remainder is only valid for certain models of section positions [Ref. 2, Definition 2.1], however, the models considered in this paper fulfill this requirement. In particular, the result also covers the case of the Cavalieri estimator (1) if the sections are equidistant with a distance *T* apart. This is treated in full in Ref. 4.

Theorem 1 (Variance of the trapezoidal estimator) Let *Y* be an *m*‐oriented three‐dimensional object, where m∈{0,1}. The variance of the trapezoidal estimator based on slices with average thickness *T* decomposes as

(4) $$\mathrm{var}\left({\hat{Q}}_{1}\right)=cT^{2m+2}+Z\left(T\right)+r\left(T\right),$$

where *c* is a *T*‐independent constant given in terms of derivatives of the area function.

The leading term $cT^{2m+2}$ is also called *extension term*, the *Zitterbewegung* Z(T) has an oscillating behaviour around 0 and is at most of size $T^{2m+2}$, and the remainder r(T) can be neglected as it decreases faster than $T^{2m+2}$ with decreasing *T*.

If we sample from the perturbed model (thus including the case of equidistant sections), the Zitterbewegung is oscillating around 0 and it is of size $T^{2m+2}$.

If we sample from the cumulative model, the Zitterbewegung decreases faster than $T^{2m+2}$ and can therefore be omitted in (4) and considered to be part of r(T).

In the equidistant case, the above smoothness considerations can be extended to fractional smoothness indices *m* leading to different orders of variance decrease than *T* ^2^ and *T* ^4^ obtained from m=0,1, respectively. For instance, a circular cylinder *Z* that is sampled with planes parallel to its axis, has an area function with smoothness m=1/2 leading to a variance decrease of order *T* ^3^. This is made precise and analysed more generally in Ref. 19 leading to a powerful extension of (4) for objects with fractional *m*. On the other hand, all convex objects with a smooth boundary and positive Gaussian curvature (satisfying some technical condition) are 1‐oriented due to [Ref. 3, Proposition 11]. These conditions exclude the cylinder *Z* and the so‐called ‘super egg’, as their boundaries have points with vanishing Gaussian curvature. It has been remarked by Cruz‐Orive and García‐Fiñana^19^, ^20^ (and in Ref. 7 for area functions derived from thick sections) that the empirical order of variance decrease in a practical application appears to be different from 2 or 4 and may even depend locally on the gap length range. The authors therefore suggest an estimation procedure for fractional *m* in the equidistant case. To extend this theory to non‐equidistant sampling is an open problem, which is in particular challenging as different gap length ranges may intermingle.

Before relating the variance behaviour presented above to the one of the Cavalieri estimator, a remark on the Zitterbewegung Z(T) is in place. The Zitterbewegung turns out to depend on the *m*th derivative of the area function. If this derivative has only one discontinuity, then the Zitterbewegung vanishes. Hence, Z(T) can only oscillate if the *m*th derivative has at least two discontinuities. However, even if the *m*th derivative has one discontinuity only, the remainder r(T) can in fact show an oscillating behaviour, though of size decreasing faster than the leading term and thus negligible for decreasing *T*; see Figure 7(left) for an example of an asymptotically vanishing Zitterbewegung.

In contrast to the behaviour given in Theorem 1 in which the variance of the trapezoidal estimator decreases as $T^{2m+2}$, the variance of the Cavalieri estimator (1) based on non‐equidistant sections most often decreases slower. In particular, if we sample from the perturbed model now excluding equidistant sections, the variance decrease is of order *T* ^2^ if *Y* is 0‐oriented, and of order *T* ^3^ if *Y* is 1‐oriented. The latter case is illustrated in Figure 1(left) for the volume estimation of the ball of radius 1. If instead we sample from the cumulative model, the variance inflation is even more pronounced. In this case, the variance decrease is of order *T* independently of *m*, which is also illustrated in Figure 1 (right) in the case m=1.

As mentioned in the Introduction, the trapezoidal estimator is constructed such that it adapts to the smoothness of the area function – just as it is the case for the Cavalieri estimator under equidistant sections. For the trapezoidal estimator, this is unfortunately only the case for weakly (*m*, 1)‐piecewise smooth functions with m≤1. If m≥2, it has a variance decreasing at a slower rate than that of the Cavalieri estimator based on equidistant sections. However, the theory of applying higher‐order quadrature rules described in Ref. 2 holds for arbitrary *m*. They consider the so‐called *m*th Newton–Cotes estimator which approximates *f* by a piecewise polynomial of order *m* (with the first Newton–Cotes estimator being the trapezoidal estimator), and in fact the variance of the *m*th Newton–Cotes estimator decreases as the variance of the Cavalieri estimator based on equidistant sections. These variance results and the unbiasedness of the estimator now require some weak technical assumptions on the process of sampling points. Since m=0 and m=1 are most relevant in practice, considering those higher‐order estimators is beyond the scope of the present paper.

## 3 ESTIMATING THE VARIANCE OF THE TRAPEZOIDAL ESTIMATOR

Concerning the estimation of the variance of the Cavalieri estimator (1) based on equidistant sections, the traditional approach is to neglect the Zitterbewegung and remainder in the decomposition (4) and thus approximate it by the extension term. There are drawbacks with this approach, as the Zitterbewegung may in fact be rather large and one risks actually underestimating the variance in particular if the number of sections is low. However, the Zitterbewegung for the trapezoidal estimator of a large class of 1‐oriented objects can never exceed the extension term by [Ref. 3, Theorem 13]. This bound holds a fortiori for the Cavalieri estimator under equidistant sections, and it is sharp without additional information as it cannot be improved if the object is a ball. The question in [Ref. 19, Section 9(4)], if an optimal data‐driven bound for the Zitterbewegung can be found, is still open. In Ref. 20, the variance (based on equidistant points only) is estimated by incorporating elements from the Zitterbewegung thus creating a more flexible estimation valid for an arbitrary number of sections. However, their estimation approach requires knowledge of the exact form of the underlying area function not always accessible in practice. Thus, in the present paper we follow the named convention and approximate the variance of the trapezoidal estimator by its (estimated) extension term. As a consequence of the above‐mentioned result [Ref. 3, Theorem 13], multiplying the estimated extension term for a 1‐oriented object by two can serve as a conservative variance estimate taking the Zitterbewegung into account.

In this section, we give estimates for the variance of the trapezoidal estimator ${\hat{Q}}_{1}$ in three different situations. The variance estimators in Definition 1 do not require any model for the sampling positions and apply also when dropouts are present. The variance estimators in Definitions 2 and 3 are based on the perturbed and cumulative model, respectively, and do not allow for dropouts. At the end of this section and in the Appendix, we will comment on model‐based variance estimators in the presence of dropouts. The following generalizations of classical variance estimators for the Cavalieri estimator have been formally derived in Ref. 3.

First, we estimate the so‐called covariogram $g\left(x\right)=\int_{-\infty }^{\infty }f\left(x+y\right)f\left(y\right)dy$ of the area function *f* as follows:

(5) $$\hat{g}\left(k\right)=\sum_{j=0}^{N-k}\mathrm{Area}\left(S_{j}\right)\mathrm{Area}\left(S_{k+j}\right)$$

for k=0,⋯,N. Intuitively, this term describes the correlation of the cross‐sectional areas. Secondly, with the notation used in Ref. 3, we need estimates of certain moments denoted $\gamma_{i,j}$. The quantity $T\gamma_{i,j}$ represents the expected *j*th power of the typical distance between an observed section and its *i*th neighbour. The way $\gamma_{i,j}$ is estimated depends on whether or not additional model assumptions are imposed on the distributions of the slice thicknesses. Without extra assumptions, one can use the estimates

(6) $${\hat{\gamma }}_{i,j}=\frac{N}{N-i+1}\sum_{k=0}^{N-i}\frac{{\left(h_{k+1}+\cdots +h_{k+i}\right)}^{j}}{h_{1}+\cdots +h_{N}}$$

for i=1,⋯,N.

The estimates in (5) and (6) are sufficient to compute variance estimates of ${\hat{Q}}_{1}$ based on the observed sections only.

Definition 1 (Model‐free variance estimation) If *Y* is a 0‐oriented object we estimate the variance of ${\hat{Q}}_{1}$ by

(7) $$\hat{\mathrm{var}}\left({\hat{Q}}_{1}\right)=\left(3\hat{g}\left(0\right)-4\hat{g}\left(1\right)+\hat{g}\left(2\right)\right)\times \frac{1}{12}{\hat{\gamma }}_{1,3}.$$

If *Y* is a 1‐oriented object we estimate the variance of ${\hat{Q}}_{1}$ by

(8) $$\begin{array}{ccc} \hat{\mathrm{var}}\left({\hat{Q}}_{1}\right) & = & \frac{\hat{g}\left(0\right)\left({\hat{\gamma }}_{2,2}-{\hat{\gamma }}_{1,2}\right)-\hat{g}\left(1\right){\hat{\gamma }}_{2,2}+\hat{g}\left(2\right){\hat{\gamma }}_{1,2}}{{\hat{\gamma }}_{1,2}{\hat{\gamma }}_{2,3}-{\hat{\gamma }}_{2,2}{\hat{\gamma }}_{1,3}}\\ & & \times \;\frac{1}{120}\left(12\;{\hat{\gamma }}_{1,5}-10\;{\left({\hat{\gamma }}_{1,3}\right)}^{2}\right). \end{array}$$

The estimators (7) and (8) are motivated by the fact that these expressions would be asymptotically unbiased for the variance if all the occurring estimates ${\hat{\gamma }}_{i,j}$ were replaced by the exact values $\gamma_{i,j}$. As the coefficients $\gamma_{i,j}$ are not known, we estimate them with the (slightly biased) quantity (6), which is sufficient in typical applications, but could be refined; see [Ref. 3, Corollary 10] for details.

Note that Definition 1 contains the equidistant setting as a special case. When all slices have the same thickness *T*, we have ${\hat{\gamma }}_{ij}={\left(iT\right)}^{j}/T=i^{j}T^{j-1}$. Hence, considering the degree of smoothness m=0, we see that the right side of (7) reduces to

(9) $$\frac{T^{2}}{12}\left(3\hat{g}\left(0\right)-4\hat{g}\left(1\right)+\hat{g}\left(2\right)\right)$$

corresponding to the standard variance estimate of the Cavalieri estimator, and, if m=1, the right side of (8) simplifies to

(10) $$\frac{T^{2}}{240}\left(3\hat{g}\left(0\right)-4\hat{g}\left(1\right)+\hat{g}\left(2\right)\right),$$

which is again the standard variance estimate of the Cavalieri estimator; see, for example, [Ref. 21, Paragraph 13.2.5] for the formulas (9) and (10).

If the sampling model for the positions of the sections is known, this distributional information can be used to compute model‐specific estimates for the moments $\gamma_{i,j}$ yielding alternatives to the estimators in Definition 1. We will now present such estimates based on the perturbed and cumulative model, and refer to Ref. 3 for their justification. Note that the formulas are only valid for sampling without dropouts.

To name the key idea: When the perturbed model is an appropriate description, the coefficients $\gamma_{i,j}$ can be estimated from three rather straightforward quantities: The average, the variance and the fourth centred moment of the thickness *h*_1_ of a typical slice, estimated by

(11) $$\hat{T}=\frac{1}{N}\sum_{k=1}^{N}h_{k},$$

and

(12) $${\hat{\theta }}_{2}=\frac{1}{N}\sum_{k=1}^{N}{\left(h_{k}-\hat{T}\right)}^{2},\;{\hat{\theta }}_{4}=\frac{1}{N}\sum_{k=1}^{N}{\left(h_{k}-\hat{T}\right)}^{4},$$

respectively.

Definition 2 (Variance estimation under the perturbed model without dropouts) If *Y* is a 0‐oriented object we estimate the variance of ${\hat{Q}}_{1}$ by

(13) $$\hat{\mathrm{var}}\left({\hat{Q}}_{1}\right)=\left(3\hat{g}\left(0\right)-4\hat{g}\left(1\right)+\hat{g}\left(2\right)\right)\times \frac{1}{12}\left({\hat{T}}^{2}+3{\hat{\theta }}_{2}\right).$$

If *Y* is a 1‐oriented object, we estimate the variance of ${\hat{Q}}_{1}$ by

(14) $$\begin{array}{ccc} \hat{\mathrm{var}}\left({\hat{Q}}_{1}\right) & = & \frac{3\hat{g}\left(0\right){\hat{T}}^{2}-\hat{g}\left(1\right)\left(4{\hat{T}}^{2}+{\hat{\theta }}_{2}\right)+\hat{g}\left(2\right)\left({\hat{T}}^{2}+{\hat{\theta }}_{2}\right)}{3{\hat{\theta }}_{2}^{2}+{\hat{\theta }}_{2}{\hat{T}}^{2}+4{\hat{T}}^{4}}\\ & & \times \;\frac{1}{60}\left({\hat{T}}^{4}+30{\hat{\theta }}_{2}{\hat{T}}^{2}+30{\hat{\theta }}_{4}-45{\hat{\theta }}_{2}^{2}\right). \end{array}$$

Here, the estimates (5), (11) and (12) were used.

If all slices have the same thickness *T*, we get $\hat{T}=T$ and ${\hat{\theta }}_{2}={\hat{\theta }}_{4}=0$, so (13) and (14) reduce to the standard estimates (9) and (10) presented above.

When the cumulative model is an appropriate description, the estimates of the coefficients $\gamma_{ij}$ are easiest stated in terms of the average (11) and the quantities

(15) $${\hat{\nu }}_{j}=\frac{1}{N}\sum_{k=1}^{N}h_{k}^{j},$$

where j=1,…,5.

Definition 3 (Variance estimation under the cumulative model without dropouts) If *Y* is a 0‐oriented object, we estimate the variance of ${\hat{Q}}_{1}$ by

(16) $$\hat{\mathrm{var}}\left({\hat{Q}}_{1}\right)=\left(3\hat{g}\left(0\right)-4\hat{g}\left(1\right)+\hat{g}\left(2\right)\right)\times \frac{1}{12}\frac{{\hat{\nu }}_{3}}{\hat{T}}.$$

If *Y* is a 1‐oriented object, we estimate the variance of ${\hat{Q}}_{1}$ by

(17) $$\begin{array}{ccc} \hat{\mathrm{var}}\left({\hat{Q}}_{1}\right) & = & \frac{\hat{g}\left(0\right)\left(2{\hat{T}}^{2}+{\hat{\nu }}_{2}\right)-\hat{g}\left(1\right)\left(2{\hat{T}}^{2}+2{\hat{\nu }}_{2}\right)+\hat{g}\left(2\right){\hat{\nu }}_{2}}{6{\hat{\nu }}_{2}^{2}{\hat{T}}^{2}-2{\hat{\nu }}_{3}{\hat{T}}^{3}}\\ & & \times \;\frac{1}{120}\left(12\;{\hat{\nu }}_{5}\hat{T}-10\;{\hat{\nu }}_{3}^{2}\right). \end{array}$$

Here, the estimates (5), (11) and (15) were used.

If the area function cannot be observed in all section planes (dropouts), the estimators in Definition 1 can still be used just disregarding all the positions of planes where the area function is unknown. However, when a model for the sampling positions is assumed, dropouts typically destroy the model assumptions; for instance, dropouts after equidistant sampling result in sampling positions that are no longer equidistant. Hence, the estimates in Definitions 2 and 3, which depend on specific models, should no longer be used. However, adapted estimators for sampling with dropouts that exploit model assumptions can still be stated. We describe them in detail in the Appendix.

It may also be noted that the estimation of certain quantities in Definitions 2 and 3 are in fact slightly biased. Exploiting the underlying model assumptions it is not difficult to obtain refined and unbiased estimators. For instance, the quantities estimated by ${\hat{\theta }}_{2}$ and ${\hat{T}}^{2}$ in (13) can be estimated slightly better by ${\hat{\theta }}_{2}N^{2}/\left(N^{2}-1\right)$ and ${\hat{T}}^{2}-{\hat{\theta }}_{2}/\left(N^{2}-1\right)$, respectively. However, this appears to be of minor importance for applications, in particular when no dropouts have occurred. In fact, in the case of no dropouts the variance estimators using the refined estimators are indistinguishable from those presented in Definitions 2 and 3. As the refined estimators come with the cost of rather complicated formulas, we have not included them here and instead recommend the estimators of the present section in microscopy applications.

## 4 SIMULATION STUDY

**FIGURE 6:**
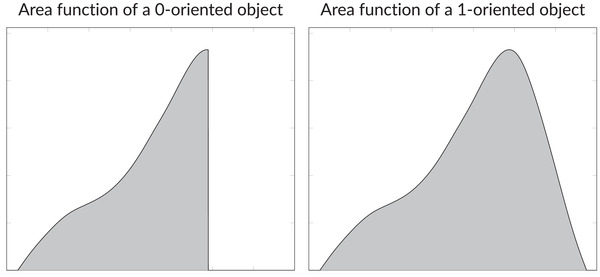
Area functions based on cubic spline interpolation of data from 18 monkey parietal lobes (right). On the left, this function is truncated to obtain an area function example of a 0‐oriented object. In the simulations, the area function on the left is rescaled in *x*‐direction in order to make the mean numbers of hitting intersections comparable

We conclude the paper by illustrating our results with a simulation study based on data from 18 monkey parietal lobes. The details of the original study have been reported in Refs. 11, 22, 23, and further details of importance for the simulation study have been covered in Johanna Ziegel's PhD thesis.^24^ In brief: 18 parietal lobes from macaque monkeys were embedded in low‐melt agarose and cut into 2.5‐mm thick slabs perpendicular to the intra‐parietal sulcus. The slabs were generated using the ‘shoebox cutter’ where the tissue is embedded together with a paper strip with 2.5‐mm marks and cut, aiming at these marks using a box‐shaped transparent cutting guide fitting the block [Ref. 18, fig. 2D–E]. This resulted in 12–15 slabs per parietal lobe. The total thickness of each resulting stack of slabs were measured using an electronic caliper. In addition, a photo (appx. 2000 pixels wide) of each stack of slabs were recorded (Figure 3). From the photos and the caliper measurements, the exact positions of each cut were easily obtained for each specimen. Subsequently, the area of the cut surface of each slab, observed under a stereomicroscope at 9× magnification, were estimated by point counts, using a uniformly randomly superimposed transparent point grid with an area per point $a=2.29\;{\text{mm}}^{2}$. An average of 645 points were counted in total across all slabs per specimen. Thus, data sets of estimated area and corresponding position of each cut surface were generated for each of the 18 specimens. Using these data points, we created 18 area functions using cubic spline interpolations. We then constructed a typical area function of a 1‐oriented object by averaging all 18 spline functions. As an example of the area function of a 0‐oriented object, we simply restricted the above area function to a smaller interval thus obtaining a single discontinuity (see Figure 6). We emphasize that spline smoothing is *not* meant as a tool to be used in real applications. It is applied here to construct fictional area functions based on real biological data to illustrate our methods, as the randomized sampling locations require that the area function can be evaluated at any point. Although created with a slightly different interpolation scheme, the area functions are very similar to the ones used in the simulation study of Ref. 10, which were based on the same underlying biological data.

As stated previously, sections resulting in 0‐oriented objects should be avoided if possible. However, sometimes – especially in archival material – histological sections may be generated from an anatomical subregion with a human‐made flat surface, due to the way the tissue block was cut from the patient, and unwisely sectioned parallel to the flat surface (the intuitive default, which we dissuade from using).

In the following analysis, we restrict our attention to the case of sections sampled from the *perturbed model without dropouts*. We refer to the Appendix for a short description in the case of dropouts.

**FIGURE 7:**
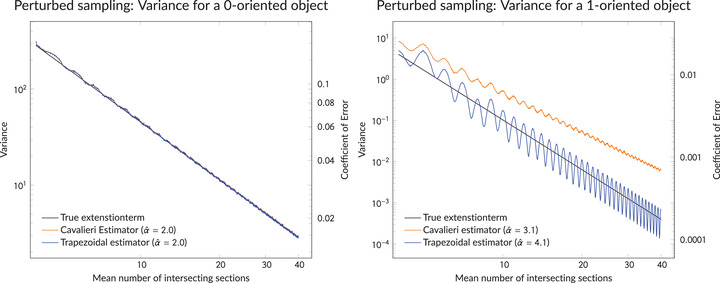
Empirical variances of the Cavalieri estimator and the trapezoidal estimator for the 0‐oriented object (left) and the 1‐oriented object (right) displayed in Figure 6. The leading term of the variance expansion in (4), that is, the extension term, is included. Note that the orange and blue curves essentially coincide in the left plot. We sample from the perturbed model using truncated normal distributions with standard deviation chosen such that the average relative deviation of the slice thickness to the intended thickness *T* is 5%. In addition, we sample without dropouts

First, we illustrate the claims of Theorem 1 and the comments following it. On a log –log  scale, Figure 7 shows the empirical variance of the Cavalieri estimator (1) and the trapezoidal estimator for volume estimation of objects with the constructed area functions against the mean number 1/T of section planes hitting the objects. We included approximate decrease rates of all estimators as $\hat{\alpha }$ (found by a least squares procedure). The empirical variances are based on 5000 Monte Carlo simulations of sections sampled from the perturbed model with truncated normal perturbations and without dropouts. As expected, we see that the variance of the Cavalieri estimator and the trapezoidal estimator both decrease as *T* ^2^ with decreasing *T* for the 0‐oriented object. Moreover, due to the fact that the area function in Figure 6 (left) for the 0‐oriented object has exactly one jump, there is no Zitterbewegung for either estimator in this case. In contrast, the trapezoidal estimator clearly shows a Zitterbewegung in the variance plot for the 1‐oriented object, since the first derivative of this area function has two discontinuities (at the boundary of the support of the area function). Moreover and most importantly, we see that the trapezoidal estimator decreases as *T* ^4^ with decreasing *T*, whereas the Cavalieri estimator decreases approximately as *T* ^3^.

In the case of sections sampled from the cumulative model, the trapezoidal estimator has a behaviour very similar to the one depicted in Figure 7 although there is no Zitterbewegung in the plot for the 1‐oriented object. That is, in this case the oscillating behaviour decreases in magnitude with decreasing *T*; for an illustration of this (although with a different area function), we refer to [Ref. 2, fig. 1b]. Moreover, the Cavalieri estimator behaves substantially worse when based on sections from the cumulative model, as it decreases as *T* for both, the 0‐ and the 1‐oriented objects.

**FIGURE 8:**
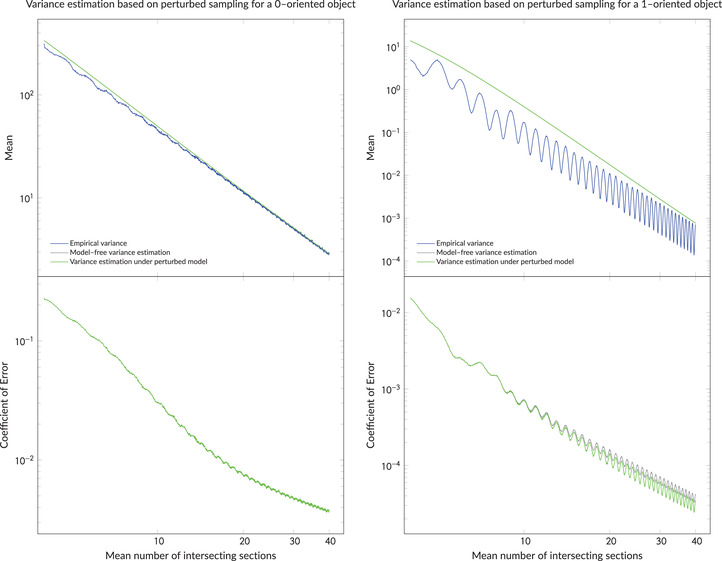
Empirical means and coefficients of error of the variance estimators presented in Definitions 1 and 2. The left plots illustrate the 0‐oriented object, and the right plots depict the 1‐oriented object. In the upper plots, the empirical variances of the trapezoidal estimators are included. Note that the grey and green curves essentially coincide in both plots to the left and in the upper right plot. The sampled sections are identical to those used in Figure 7. We emphasize again that the coefficient of error in this figure describes the variability of the *variance* estimates and not of the volume estimates

We now apply the results of the previous section on variance estimation to our constructed area functions and the newly simulated data. More precisely, based on the 5000 Monte Carlo simulations described above, Figure 8 shows (on a log –log  scale) the empirical means and coefficients of error of the trapezoidal variance estimators given in Definitions 1 and 2 for the 0‐oriented object (left) and the 1‐oriented object (right). The coefficient of error is here defined as the empirical standard deviation of the variance estimator divided by the extension term (and describes the variability of the *variance* estimates and not of the volume estimates).

We see that the variance estimators in Definitions 1 and 2 both overestimate the actual variance for either area function. However, in accordance with the comments following Definition 1, these biases are insignificant when the mean number of section planes hitting the object is large – that is, when the average thickness *T* of a slice is small. Furthermore, the estimators in Definitions 1 and 2 are essentially identical for 0‐oriented objects, both in terms of mean and coefficient of error (equivalently standard deviation), whereas for the 1‐oriented object the model‐specific estimator from Definition 2 appears to outperform the general estimator from Definition 1 in the sense that it has a lower coefficient of error.

## CONFLICT OF INTEREST

The authors declare that there is no conflict of interest that could be perceived as prejudicing the impartiality of the research reported.

## Figures and Tables

**FIGURE A.1:**
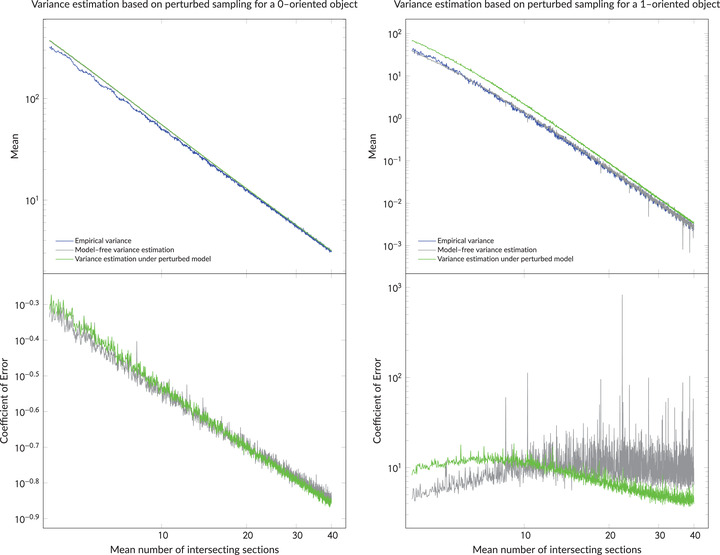
Empirical means and coefficients of error of the variance estimators presented in Definitions 1 and A.1 based on perturbed sampling with a dropout probability of 2.5%. The left plots illustrate the 0‐oriented object, and the right plots depict the 1‐oriented object. In the upper plots, the empirical variances of the trapezoidal estimators are included. Note that the grey and green curves essentially coincide in the upper left plot

## APPENDIX A ALLOWING FOR DROPOUTS

The purpose of this Appendix is to introduce variance estimators in the spirit of Definitions 2 and 3 which allow for dropouts in the sampling model. In the scenario where the area of certain section profiles cannot be determined, the variance estimators of Definition 1 can still be applied whereas those of Definitions 2 and 3 cannot. However, it is possible to exploit the underlying model to construct new variance estimators taking the dropouts into account. There are two major advantages of using the model‐specific variance estimators presented below: One is the fact that the general estimator for 1‐oriented objects given in (8) can in fact be negative if dropouts are present, and this has not been observed in simulations for the model‐based estimators given in Definitions A.1 and A.2. The second reason is that the estimators based on the underlying model appear to be much more robust than the general estimators (see Figure A.1).

To construct the new range of estimators, we have to make additional assumptions: First, we assume that section planes (or at least their areas) are dropped independently of each other with the same probability p∈[0,1). Secondly, we assume that the thickness of *all initial* slices can be measured, that is, also the ones which contain a section profile with unobserved area.

We formalize this now. There are a number of initial cross sections ${\tilde{S}}_{0},{\tilde{S}}_{1},\cdots ,{\tilde{S}}_{M}$ of the object *Y*, and we assume that we observe all the associated slice thicknesses ${\tilde{h}}_{1},{\tilde{h}}_{2},\cdots ,{\tilde{h}}_{M}$. For some of these cross sections, the area cannot be measured, resulting in the sections $S_{0},S_{1},\cdots ,S_{N}$ (where N≤M) with observable areas $\mathrm{Area}\left(S_{0}\right),\mathrm{Area}\left(S_{1}\right),\cdots$, $\mathrm{Area}(S_{N})$, where the first and last of them are zero (see Figure 2). Furthermore, we have the corresponding distances $h_{1},\cdots ,h_{N}$ between these sections. Thus, any $h_{i}$ is in fact a sum of one or more of the known initial slice thicknesses ${\tilde{h}}_{j}$. The distances $h_{1},h_{2},\cdots ,h_{N}$, combined with the known areas are used to estimate the volume of *Y* via the trapezoidal estimator (2). To compute the model‐specific variance estimators, we additionally use ${\tilde{h}}_{1},{\tilde{h}}_{2},\cdots ,{\tilde{h}}_{M}$, that is, the thicknesses of all slices, no matter if the areas are observed or not. In particular, M−N measures the number of dropped slices, and consequently M=N and ${\tilde{h}}_{i}=h_{i}$ if no dropouts occur. In the latter case, the variance estimators given below simplify to those of Definitions 2 and 3, respectively.

A typical example of this scenario would be serial sections of a tissue, where one can measure at least approximately the distances of neighbouring section planes ‐ ‐ for example, from a photo of the original tissue slabs edge with a superimposed ruler – but where not all areas in sections can be determined for instance due to staining problems, distortion or loss of one or more histological sections due to sectioning artefacts, or similar. These assumptions allow us to use ${\tilde{h}}_{i}$ in the following estimates.

With the notation above at hand,

(A.1)
$$\tilde{p}=\frac{M-N}{M+1}$$

is an estimate of the probability *p* of *not* observing the area of a given section (we refer to it as the *thinning probability*). Similarly

(A.2)
$$\tilde{sp}=\frac{N+1}{M+1}$$

is an estimate of (1−p) (sp is short for *survival probability*). Clearly, $\tilde{sp}$ can be computed from $\tilde{p}$ and vice versa, but for notational convenience, we have introduced both estimators.

To compute the model‐specific estimators, we need the estimators (11), (12) and (15) but based on all slices, including the ones with unobserved area, that is, based on the slices before thinning. With the new notation,

(A.3)
$$\tilde{T}=\frac{1}{M}\sum_{k=1}^{M}{\tilde{h}}_{k}$$

estimates the average slice thickness,

(A.4)
$${\tilde{\theta }}_{2}=\frac{1}{M}\sum_{k=1}^{M}{\left({\tilde{h}}_{k}-\tilde{T}\right)}^{2}\;\text{and}\;{\tilde{\theta }}_{4}=\frac{1}{M}\sum_{k=1}^{M}{\left({\tilde{h}}_{k}-\tilde{T}\right)}^{4}$$

estimate the variance and the fourth centred moment of the typical slice thickness under the perturbed model, and

(A.5)
$${\tilde{\nu }}_{j}=\frac{1}{M}\sum_{k=1}^{M}{\left({\tilde{h}}_{k}\right)}^{j}$$

for j=1,⋯,5 estimate moments of the typical slice thickness under the cumulative model.
Definition A.1
(Variance estimation under the perturbed model with dropouts) Using the notations (5), (A.1), (A.2), (A.3) and (A.4) let

(A.6)
$$\begin{array}{cc} {\tilde{\gamma }}_{1,3}^{p} & ={\tilde{T}}^{2}+3{\tilde{\theta }}_{2}+{\tilde{T}}^{2}6\frac{\tilde{p}}{{\tilde{sp}}^{2}}, \end{array}$$

(A.7)
$$\begin{array}{ccc} {\tilde{C}}_{0}^{p} & = & {\tilde{T}}^{2}\frac{3+\tilde{p}}{\tilde{sp}},\;{\tilde{C}}_{1}^{p}=\tilde{sp}{\tilde{\theta }}_{2}+{\tilde{T}}^{2}\frac{4+2\tilde{p}}{\tilde{sp}},\\ & & \;{\tilde{C}}_{2}^{p}=\tilde{sp}{\tilde{\theta }}_{2}+{\tilde{T}}^{2}\frac{1+\tilde{p}}{\tilde{sp}}, \end{array}$$

(A.8)
$$\begin{array}{cc} {\tilde{\mathrm{denom}}}^{p} & =3\tilde{sp}{\tilde{\theta }}_{2}^{2}+{\tilde{T}}^{2}{\tilde{\theta }}_{2}\frac{1+10\tilde{p}+{\tilde{p}}^{2}}{\tilde{sp}}+4{\tilde{T}}^{4}\frac{1+\tilde{p}+{\tilde{p}}^{2}}{{\tilde{sp}}^{3}}, \end{array}$$

(A.9)
$$\begin{array}{ccc} {\tilde{\mathrm{diff}}}^{p} & = & \frac{{\tilde{T}}^{4}}{60}+\frac{{\tilde{\theta }}_{4}}{2}-\frac{3{\tilde{\theta }}_{2}^{2}}{4}+{\tilde{T}}^{2}{\tilde{\theta }}_{2}\frac{1+4\tilde{p}+{\tilde{p}}^{2}}{2{\tilde{sp}}^{2}}\\ & & +\;{\tilde{T}}^{4}\frac{2\tilde{p}+5{\tilde{p}}^{2}+2{\tilde{p}}^{3}}{{\tilde{sp}}^{4}}. \end{array}$$

If *Y* is a 0‐oriented object, we estimate the variance of ${\hat{Q}}_{1}$ by

(A.10)
$$\tilde{\mathrm{var}}\left({\hat{Q}}_{1}\right)=\left(3\hat{g}\left(0\right)-4\hat{g}\left(1\right)+\hat{g}\left(2\right)\right)\times \frac{1}{12}{\tilde{\gamma }}_{1,3}^{p}.$$

If *Y* is a 1‐oriented object, we estimate the variance of ${\hat{Q}}_{1}$ by

(A.11)
$$\tilde{\mathrm{var}}\left({\hat{Q}}_{1}\right)=\frac{\hat{g}\left(0\right){\tilde{C}}_{0}^{p}-\hat{g}\left(1\right){\tilde{C}}_{1}^{p}+\hat{g}\left(2\right){\tilde{C}}_{2}^{p}}{{\tilde{\mathrm{denom}}}^{p}}\times {\tilde{\mathrm{diff}}}^{p}.$$

Definition A.2
(Variance estimation under the cumulative model with dropouts) Using the notations (5), (A.1), (A.2), (A.3) and (A.5) let

(A.12)
$$\begin{array}{cc} {\tilde{\gamma }}_{1,3}^{c} & =\frac{{\tilde{\nu }}_{3}}{\tilde{T}}+6{\tilde{\nu }}_{2}\frac{\tilde{p}}{\tilde{sp}}+{\tilde{T}}^{2}6\frac{{\tilde{p}}^{2}}{{\tilde{sp}}^{2}}, \end{array}$$

(A.13)
$$\begin{array}{ccc} {\tilde{C}}_{0}^{c} & = & {\tilde{\nu }}_{2}+{\tilde{T}}^{2}\frac{2+2\tilde{p}}{\tilde{sp}},\;{\tilde{C}}_{1}^{c}=2{\tilde{\nu }}_{2}+{\tilde{T}}^{2}\frac{2+4\tilde{p}}{\tilde{sp}},\;\\ & & {\tilde{C}}_{2}^{c}={\tilde{\nu }}_{2}+{\tilde{T}}^{2}\frac{2\tilde{p}}{\tilde{sp}}, \end{array}$$

(A.14)
$$\begin{array}{cc} {\tilde{\mathrm{denom}}}^{c} & =\frac{6{\tilde{\nu }}_{2}^{2}{\tilde{T}}^{2}}{\tilde{sp}}-\frac{2{\tilde{\nu }}_{3}{\tilde{T}}^{3}}{\tilde{sp}}+12{\tilde{\nu }}_{2}{\tilde{T}}^{4}\frac{\tilde{p}}{{\tilde{sp}}^{2}}+12{\tilde{T}}^{6}\frac{{\tilde{p}}^{2}}{{\hat{sp}}^{3}}, \end{array}$$

(A.15)
$$\begin{array}{ccc} {\tilde{\mathrm{diff}}}^{c} & = & \frac{\tilde{T}{\tilde{\nu }}_{5}}{10}-\frac{{\tilde{\nu }}_{3}^{2}}{12}+\frac{\left({\tilde{T}}^{2}{\tilde{\nu }}_{4}+\tilde{T}{\tilde{\nu }}_{3}{\tilde{\nu }}_{2}\right)\tilde{p}}{\tilde{sp}}+\frac{\left(5{\tilde{T}}^{3}{\tilde{\nu }}_{3}+6{\tilde{T}}^{2}{\tilde{\nu }}_{2}^{2}\right){\tilde{p}}^{2}}{{\tilde{sp}}^{2}}\\ & & +\;18{\tilde{T}}^{4}{\tilde{\nu }}_{2}\frac{{\tilde{p}}^{3}}{{\tilde{sp}}^{3}}+9{\tilde{T}}^{6}\frac{{\tilde{p}}^{4}}{{\tilde{sp}}^{4}}. \end{array}$$

If *Y* is a 0‐oriented object, we estimate the variance of ${\hat{Q}}_{1}$ by

(A.16)
$$\tilde{\mathrm{var}}\left({\hat{Q}}_{1}\right)=\left(3\hat{g}\left(0\right)-4\hat{g}\left(1\right)+\hat{g}\left(2\right)\right)\times \frac{1}{12}{\tilde{\gamma }}_{1,3}^{c}.$$

If *Y* is a 1‐oriented object, we estimate the variance of ${\hat{Q}}_{1}$ by

(A.17)
$$\tilde{\mathrm{var}}\left({\hat{Q}}_{1}\right)=\frac{\hat{g}\left(0\right){\tilde{C}}_{0}^{c}-\hat{g}\left(1\right){\tilde{C}}_{1}^{c}+\hat{g}\left(2\right){\tilde{C}}_{2}^{c}}{{\tilde{\mathrm{denom}}}^{c}}\times {\tilde{\mathrm{diff}}}^{c}.$$

Based on 5000 Monte Carlo simulations of sections sampled from the perturbed model with a thinning probability of 2.5%, we applied the variance estimators of Definitions 1 and A.1 to the area functions of the previous section. The result, depicted in log –log  scales in Figure A.1, shows that the model‐based estimators have a tendency to overestimate the variance slightly and thus produce a conservative variance estimate when compared to the general estimators of Definition 1. However, the estimates of Definition 4 clearly behave more robust (in particular for 1‐oriented objects) with a lower coefficient of error when using a mean number of sections greater than approximately 10.
